# Celecoxib-Induced Cytotoxic Effect Is Potentiated by Inhibition of Autophagy in Human Urothelial Carcinoma Cells

**DOI:** 10.1371/journal.pone.0082034

**Published:** 2013-12-09

**Authors:** Kuo-How Huang, Kuan-Lin Kuo, I-Lin Ho, Hong-Chiang Chang, Yuan-Ting Chuang, Wei-Chou Lin, Ping-Yi Lee, Shih-Chen Chang, Chih-Kang Chiang, Yeong-Shiau Pu, Chien-Tso Chou, Chen-Hsun Hsu, Shing-Hwa Liu

**Affiliations:** 1 Graduate institute of Toxicology, College of Medicine, National Taiwan University, Taipei, Taiwan; 2 Department of Urology, National Taiwan University Hospital, Taipei, Taiwan; 3 Department of Pathology, National Taiwan University Hospital, Taipei, Taiwan; 4 Department of Integrated Diagnostics and Therapeutics, National Taiwan University Hospital, Taipei, Taiwan; 5 Department of Chemical Engineering, College of Engineering, National Taiwan University, Taipei, Taiwan; Wayne State University School of Medicine, United States of America

## Abstract

Celecoxib, a cyclooxygenase-2 (COX-2) inhibitor, can elicit anti-tumor effects in various malignancies. Here, we sought to clarify the role of autophagy in celecoxib-induced cytotoxicity in human urothelial carcinoma (UC) cells. The results shows celecoxib induced cellular stress response such as endoplasmic reticulum (ER) stress, phosopho-SAPK/JNK, and phosopho-c-Jun as well as autophagosome formation in UC cells. Inhibition of autophagy by 3-methyladenine (3-MA), bafilomycin A1 or ATG7 knockdown potentiated celecoxib-induced apoptosis. Up-regulation of autophagy by rapamycin or GFP-LC3B-transfection alleviated celecoxib-induced cytotoxicity in UC cells. Taken together, the inhibition of autophagy enhances therapeutic efficacy of celecoxib in UC cells, suggesting a novel therapeutic strategy against UC.

## Introduction

Urothelial carcinoma (UC) of bladder is the fourth most common cancer in men and ninth most common cancer in women in the United States. It was estimated to account for over 70,000 new cases and 14,000 deaths in the United Stated in 2010 [1]. Advanced bladder UC has always been a devastating disease [2]. Cisplatin-based chemotherapy is the standard treatment for patients with metastatic UC [3], [4]; however, despite regimens such as the cisplatin, gemcitabine or paclitaxel combination, the overall response rates vary between 40% and 65% [5], [6]. Most patients eventually die of metastatic disease and the overall median survival is about 1 year [2]. Moreover, patients suffer chemotherapy-related side effects or toxicities during the process [4], [6]. Intense efforts have focused on the development of active agents to improve the therapeutic efficacy. It is imperative to develop new therapeutic strategy to prolong survival and minimize chemotherapy-related morbidity [2].

Celecoxib is a selective inhibitor of cyclooxygenase-2 (COX-2) and is widely used for anti-inflammation or pain control. Selective COX-2 inhibitor has been reported to elicit anti-proliferative response in various tumors [7], [8], [9], [10], [11] including urinary bladder cancer [8], [9], [12], [13], [14]; however, the detail effects and mechanisms of celecoxib on UC cells have not been fully explored. Several mechanisms have been proposed in other tumor models such as induction of cell cycle arrest, mitochondria-mediated pathway, Akt phosphorylation inhibition, endoplasmic reticulum (ER) stress, and autophagy [15], [16], [17], [18], [19], [20].

Autophagy is a process of cell destruction whereby cytoplasmic proteins and organelles are sequestered in vacuoles and delivered to lysosomes for degradation, which supports metabolism for tumor growth [21], [22]. Some anti-cancer drugs have been reported to induce autophagy and apoptosis [23], [24]. Targeting autophagy to sensitize cancers may be an effective therapeutic strategy to conquer drug resistance [23]. Therefore, we hypothesize the interference of autophagy can enhance the celecoxib-induced cytotoxicity in bladder UC cells. In this study, we try to investigate the role of autophagy in celecoxib-induced cytotoxicity in human bladder UC cells.

## Materials and Methods

### Cell Culture

NTUB1 cell line, kindly provided from Dr. Yeong-Shiau Pu (Department of Urology, National Taiwan University Hospital, Taipei, Taiwan), was derived from the surgical specimen of a 70-year-old female patient with high grade transitional bladder cell carcinoma and were reported to be tumorigenic in a xenograft model [16], [25], [26], [27], [28], [29], [30], [31], [32]. T24 cell line, obtained from the Bioresource Collection and Research Center (BCRC, Hsinchu, Taiwan), was derived from a highly malignant grade III human urinary bladder carcinoma [33]. The cells were maintained at 37°C with 5% CO_2_ in RPMI-1640 medium (NTUB1 cells) or Dulbecco's Modified Eagle Medium (T24 cells) supplemented with 10% fetal bovine serum (FBS), 100 units/ml penicillin and 1 µmg/ml streptomycin. The cell culture media and supplements were purchased from Invitrogen (Carlsbad, CA, USA).

### Reagents and Antibodies

Celecoxib pure compound was provided by Pfizer (New York, NY, USA). ZVAD-FMK (Z-Val-Ala-Asp(OMe)-CH2F), 3-MA, rapamycin, bafilomycin A1 and LM-1685 were obtained from Merck Calbiochem (Darmstadt, Germany). Antibodies against cleaved caspase-3, cleaved caspase-7, cleaved PARP, phospho-SAPK/JNK (Thr183/Tyr185), phospho-c-Jun (Ser73), ATF-4, phospho-eIF2α (Ser51), autophagy-related protein 5, 12 (Atg5, 12), and microtubule-associated protein light chain 3 B (LC3B), for immunoblotting analysis or immunofluorescence staining were purchased from Cell Signaling Technology (Danvers, MA, USA). Moreover, α-tubulin antibody was purchased from GeneTex (Irvine, CA, USA), GAPDH and β-actin antibodies were purchased from Santa Cruz Biotechnology (Santa Cruz, CA, USA). Other chemicals and reagents all obtained from Sigma-Aldrich (St. Louis, MO, USA) or Serva (Heidelberg, Germany).

### Cell Viability and Flow Cytometry (FACS) for Apoptosis Assay

Celecoxib, ZVAD-FMK, 3-MA, bafilomycin A1, rapamycin, LM-1685 or DMSO (Mock, as non-treated control) were diluted in the culture media promptly before exposing to cells. Following the treatments, 3-(4,5-dimethylthiazol-2-yl)-2,5-diphenyl tetrazolium (MTT, Sigma-Aldrich) assay were performed to detect cell viabilities according to the methods described previously [4], [6]. For apoptosis assay, the cells were harvested as described earlier and analyzed with Becton Dickinson LSR II flow cytometry (BD Bioscience, San Jose, CA, USA) [4], [6].

### Immunoblotting

Immunoblotting analysis was performed as described previously [26]. Briefly, the protein extractions from cell lysates resolved by SDS-polyacrylamide gel electrophoresis (SDS-PAGE) and transferred to polyvinylidene fluoride (PVDF) membrane (GE Healthcare, Piscataway, NJ, USA). Then the membranes were immunoblotted with various primary antibodies. Therefore, after the horseradish peroxidase (HRP)-conjugated secondary antibodies (Cell Signaling Technology) applied, antibody bound-membranes were visualized by western chemiluminescent HRP substrate (Millipore, Billerica, MA, USA). The levels of target proteins were quantified by Western blot using Image J (NIH, USA) and normalized to each internal control. The values indicated under each band were relative to each control that normalized to 1.0 and demonstrated the changes of protein expression levels.

### Immunofluorescence Staining

UC cells were grown on the glass cover slides for 24 h with complete medium. After various treatments, the cells were washed with PBS one time and fixed with 4% paraformaldehyde in PBS for 10 min at room temperature. Then, the cells were permeabilized with 0.1% Triton X-100 in PBS for 5 min followed by washing 3 times with PBS, then incubated in 10% FBS in PBS for 30 min for blocking. The primary antibodies were combined with appreciate dilution ratios in blocking buffer and added onto the cover glass and placed at 4°C overnight. After being washed with PBS, the cells were incubated with proper fluorescence conjugated-secondary antibody for 2 h. Cells were washed with PBS and visualized with fluorescence microscope and Zeiss LSM 510 laser-scanning confocal microscope. Nucleus was visualized by Hoechst (Sigma-Aldrich) staining.

### Acidic Lysosome/Autophagosome Staining

UC cells were cultured with complete medium at 37°C for 24 h then exposed to various treatments. The culture media were replaced with fresh complete medium containing 50 nM LysoTracker Red® (Invitrogen) at 37°C for 1.5 h. After washing with PBS twice, the cells were observed with fluorescence microscope. For quantification, the drug treated-cells were harvested by trypsin-EDTA solution (Invitrogen) before LysoTracker Red® incubation, and then analyzed with Becton Dickinson LSR II flow cytometry.

### Transfection of plasmids into UC cells

Cells were cultured at 37°C to the density of 30-40% before transfection. GFP-LC3B and GFP control plasmids were transfected into cells by lipofectamine 2000 (Invitrogen) according to the manufacturer's instructions. The transfected cells were exposed to celecoxib or co-treated with other compounds in various concentrations. Then the cells were collected for immunoblotting and cell viability assays.

### Knockdown of ATG7 by using siRNA

In order to knockdown ATG7, NTUB1 and T24 cells were transfected with 10 nM small interfering RNA (siRNA) against ATG7 (Thermo Scientific Dharmacon, Lafayette, CO) or 10 nM nonsilencing scramble siRNA (as control) with using DharmaFECT 1 transfection reagent (Thermo Scientific Dharmacon) according to the instructions in the manufacturer's manual. After 24 h, the media was replaced and the cells were incubated for an additional 24 h with 80 µM celecoxib or DMSO (Mock, as non-treated control) in complete medium for MTT viability assay.

### Statistical Analysis

The GraphPad Prism® 5 software was used to perform all data analysis. All data were expressed as mean ± SD and analyzed by one-way ANOVA followed by Bonferroni post hoc test, with values of *P*<0.05 considered statically significant.

## Results

### Celecoxib induces Autophagosome formation in human UC cells

**Figure 1:**
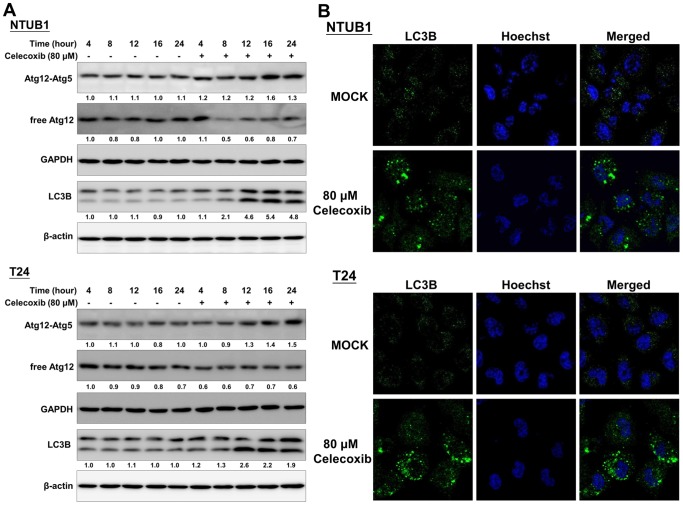
Celecoxib induces autophagy in human UC cells. (**A**) NTUB1 and T24 cells were treated with 80 µM celecoxib for 24 h. Cell lysates were harvested at six time points (4, 8, 12, 16 and 24 h) and analyzed by Western blotting using specific antibodies of anti-ATG12, anti-ATG12-ATG5 conjugate, and anti-LC3B antibodies. (**B**) UC cells were exposed to DMSO (Mock, as non-treated control) and celecoxib (80 µM) for 24 h. The LC3B immunofluorescence staining and Hoechst staining were observed by confocal microscope. Results shown are representative of at least three independent experiments.

Celecoxib has been reported to induce ER stress [16], [20]. First, we observed treatment of NTUB1 and T24 cells with 80 µM celecoxib could induce activations of stress-related molecules such as phospho-eIF2α, ATF-4, phospho-SAPK/JNK and phospho-c-Jun (Fig. S1). Autophagy is known to play the important roles in coping with multiple forms of cellular stress, including nutrient or growth factor deprivation, hypoxia, reactive oxygen species, DNA damage, and damaged organelles [21], [22], [34], [35]. In the process of autophagy, there are two ubiquitin-like conjugation systems, Atg12-Atg5 and LC3B, which are required for the initiation and expansion of autophagosomal membrane [36], [37]. To examine whether celecoxib could induce autophagy, we then analyzed the effect of celecoxib on endogenous Atg12-Atg5 conjugate in UC cells. Interestingly, we found that the levels of Atg12-Atg5 conjugate increased significantly after treatment of celecoxib (Fig. 1A). Meanwhile, celecoxib induced activation of LC3B, a marker of autophagosome formation [38] (Fig. 1A). Immunofluorescent Hoechst and LC3B staining also showed the staining pattern changed from diffuse to more punctate in cytoplasm, which meant LC3B was bound to the autophagosome [38] (Fig. 1B).

### Celecoxib induces the LysoTracker-positive staining in human UC cells

**Figure 2:**
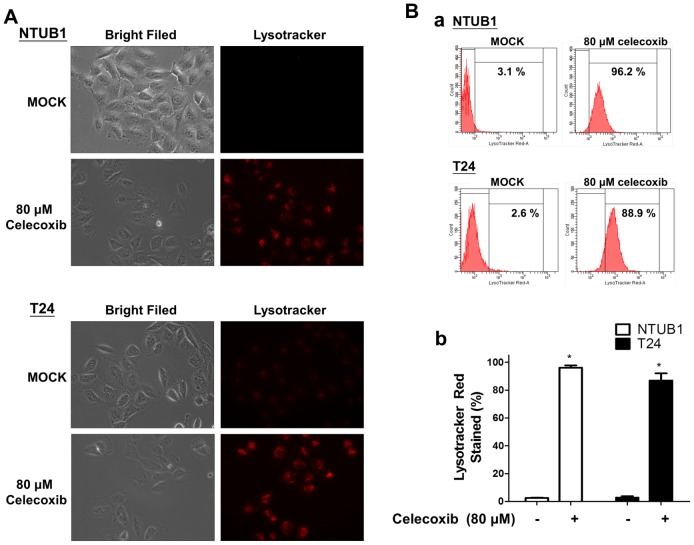
Celecoxib induces LysoTracker staining in human UC cells. (**A**) UC cells were exposed to DMSO (Mock, as non-trated control) and celecoxib (80 µM) for 24 h and then stained with 50 nM LysoTracker Red at 37°C for 1.5 h. Fluorescent images were obtained with Zeiss Axiovert 200x microscope. (**B-a**) Flowcytometry showed celecoxib treatment induced the proportion of LysoTracker-positive cells to increase. (**B-b**) Quantitative analysis of LysoTracker-positive cells following 80 µM celecoxib treatment was presented. Data are presented as means ± SD. Results shown are representative of at least three independent experiments.

Autophagy consists of at least a three steps: autophagosome formation for engulfing the cytosolic components, lysosome-autophagosome fusion, and lysosomal degradation. Autophagosome is formed de novo to sequester cytoplasm and to fuse with the lysosomes, where the constituents could be degraded and recycled [39], [40], [41]. As shown in Fig. 2A, celecoxib treatment induced prominent LysoTracker Red signals in UC cells. The red dye of LysoTracker staining indicated the presence of acidic lysosome/autophagosome in cells. Flow cytometry for quantitatively analysis demonstrated significantly increased proportion of LysoTracker-positive cells after celecoxib treatment (Fig. 2B) [15], [17].

### Autophagy inhibition enhance celecoxib-induced apoptosis in human UC cells

In the present study, celecoxib actually induced the accumulation of autophagosomes in UC cells. In most cases, autophagic cell death is cell death with autophagy rather than cell death by autophagy. Unless autophagy inhibition could alter the fate of the cell and reduce cell death, then cell death was to occur by autophagy. Moreover, autophagic cell death is theoretically caspase-independent; inhibition of caspases activation would not exert significant effects on cell death in case of cell death by autophagy.

First, the effects of celecoxib in different concentrations (0, 20, 40, 60 and 80 µM) on UC cells to induce apoptosis and autophagy are shown in Fig. S2. Celecoxib induced the activations of LC3B and cleaved caspase-7 concomitantly in a dose dependent manner;however, there still exist controversies for the role of autophagy in cancer therapy. It was recently suggested that autophagy plays different roles (defensive or destructive) in cancer therapy [42].

**Figure 3:**
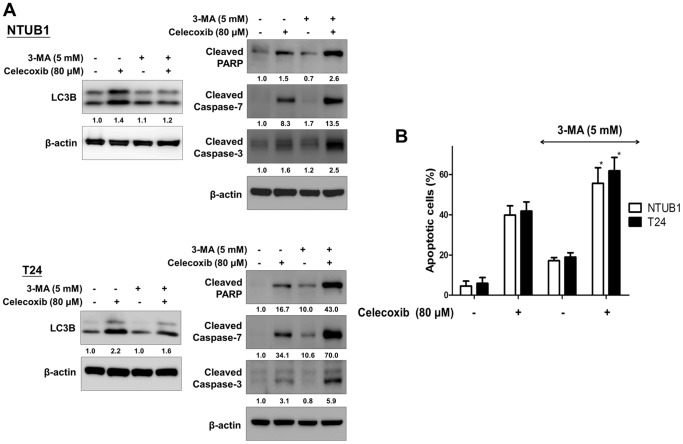
Inhibition of autophagy by 3-MA potentiates celecoxib-induced apoptosis in human UC cells. UC cells were pretreated with 5-MA for 1.5 h prior to the celecoxib (80 µM) treatment for 24 h. (**A**) The cell lysates were analyzed by immunoblotting with antibodies against LC3B, cleaved caspase-3, cleaved caspase-7, and cleaved PARP. (**B**) Apoptotic cells were analyzed by FACS flow cytometry with propidium iodide (PI) and annexin V-FITC staining. Quantitative analysis of total apoptosis (early and late) population were presented as means ± SD. *: p<0.05 as statistically significant compared with celecoxib alone. Results shown are representative of at least three independent experiments.

To investigate the role of autophagy plays in celecoxib-induced apoptosis, we first examined the apoptotic effect of celecoxib in combination with 3-methyladenine (3-MA), an autophagy inhibitor by blocking autophagosome formation via the inhibition of type BI Phosphatidylinositol 3-kinases (PI-3K) [43]. As shown in Figure 3A, 3-MA (5 mM) effectively suppressed celecoxib-induced LC3B activation and enhanced celecoxib-induced cleavage of caspase 3, 7 and PARP in NTUB1 and T24 cells. Inhibition of autophagy by 3-MA significantly potentiated celecoxib-induced apoptosis in human UC cells (Fig. 3B).

Similarly, bafilomycin A1 is a specific inhibitor of the vacuolar type H (+)-ATPase (V-ATPase) in cells, and inhibits the acidification of organelles containing this enzyme, such as lysosomes and endosomes. Bafilomycin A1 also blocks the turnover of autophagosomes [44]. To further validate the defensive role of autophagy in clecoxib-induced apoptosis, we co-treated UC cells with celecoxib (80 µM) and bafilomycin A1 (5 nM) for 24 h and found that bafilomycin A1 also potentiated celecoxib-induced apoptosis (Fig. S3A).

We further suppressed autophagy by ATG7 siRNA transfection in UC cells. The celecoxib-induced cytotoxicity was significantly potentiated by ATG7 knockdnown (Fig S3B) compared to scramble siRNA transfection. All these findings consistently indicated that autophagy inhibition enhances celecoxib-induced apoptosis in human UC cells. To sum up, we observed that autophagy inhibition potentiated cell apoptosis; therefore, we assume that autophagy is considered to be a pro-survival pathway in celecoxib-induced apoptosis.

Furthermore, we use the pan-caspase inhibitor (ZVAD-FMK) to clarify the role of autophagic cell death for celecoxib. We found ZVAD-FMK significantly decreased celecoxib-induced apoptotsis in T24 and NTUB1 cells, suggestting that activations of caspases are involved in celecoxib-mediated apoptosis (data not shown). This phenomenon implies that celecoxib induces cell death with autophagy rather than by autophagy.

### mTOR inhibitor, rapamycin alleviate celecoxib-induced UC cell apoptosis

**Figure 4:**
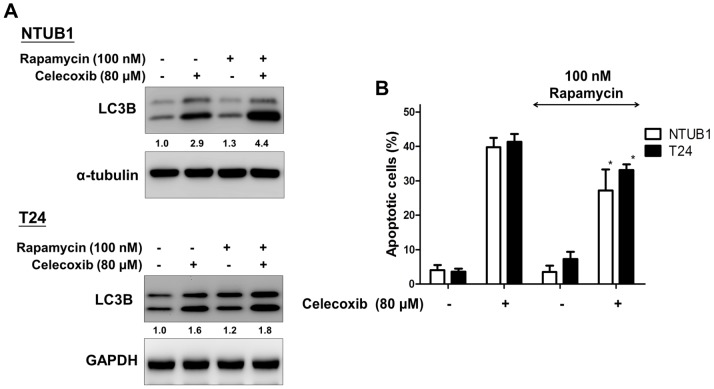
Rapamycin, an mTOR inhibitor alleviates celecoxib-induced apoptosis via enhancement of autophagy. NTUB1 and T24 cells were co-treated with celecoxib (80 µM) and rapamycin (100 nM) for 24 h. (**A**) The cell lysates were analyzed by immunoblotting with antibodies against LC3B. (**B**) Apoptotic cells were analyzed by FACS flow cytometry with propidium iodide (PI) and annexin V-FITC staining. Data are presented as the mean ± SD (n = 3). *: p<0.05 as statistically significant compared with celecoxib alone.

Mammalian target of rapamycin (mTOR) has been known as a key mediator of autophagy [45]. We then tested the combinative effect of rapamycin and celecoxib on UC cells. Immunobloting analysis by antibodies against LC3B showed that rapamycin (100 nM) effectively enhances celecoxib (80 µM)-induced LC3B activation in UC cells (Fig. 4A). Flow cytometry analysis showed that rapamycin could also alleviate celecoxib-induced UC cell apoptosis (Fig. 4B).

### LC3B overexpression alleviate celecoxib-induced UC cell apoptosis

**Figure 5:**
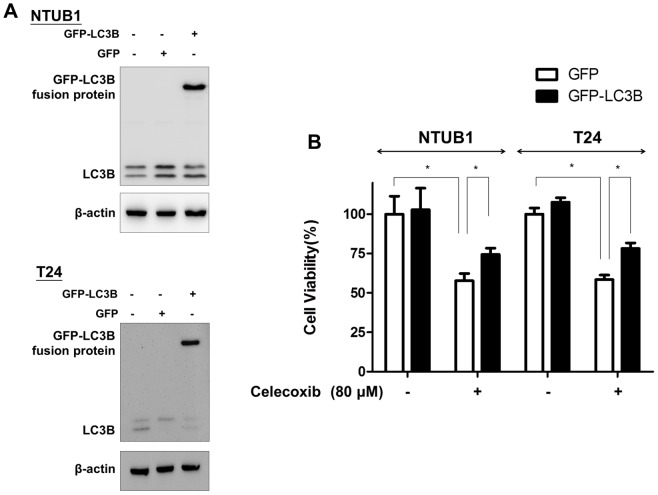
LC3B transfection alleviates celecoxib-induced cytotoxicity. GFP-LC3B and GFP control plasmids were transfected into UC cells by lipofectamine. (**A**) The transfected UC cells expressed the high level of GFP-LC3B protein in NTUB1 and T24 cells. (**B**) The celecoxib-induced cytotoxicity measured by MTT assay was rescued in GFP-LC3B-transfected cells after celecoxib treatment. *: p<0.05 as statistically significant. Results shown are representative of at least three independent experiments.

The green fluorescent protein (GFP)-tagged LC3B expressing cells were used to demonstrate induction of autophagy [38]. By using this method, GFP-LC3B and GFP control plasmids were transfected into UC cells by lipofectamine. The transfected UC cells expressed the high level of GFP-LC3B protein (Fig. 5A). The celecoxib-induced cytotoxicity was rescued in GFP-LC3B transfected cells (Fig. 5B). These data prove that the up-regulation of autophagy by rapamycin or GFP-LC3B transfection could alleviate celecoxib-induced cytotoxicity in UC cells.

### LM-1685, a celecoxib analogue and COX-2 inhibitor, does not significantly induce ER stress and autophagy in human UC cells

LM-1685, a celecoxib analogue, is also a COX-2 inhibitor. LM-1685 did not induce the expression of ER stress-related molecules and LC3B activation in UC cells (Fig. S4). We assume that celecoxib-induced cytotoxicity, ER stress and autophagy may not be through the mechanism of COX-2 inhibition.

## Discussion

Selective COX-2 inhibitor has been reported to elicit anti-proliferative responses in various tumors [7], [8], [9], [10], [11]. The exact anti-tumor mechanism of celecoxib on UCs remains elusive. In this study, we found LM-1685, a celecoxib analogue and COX-2 inhibitor, could not induce apoptosis in UC cells via inductions of ER stress and concomitant autophagy activation as celecoxib did. COX-2 inhibition may not the essential mechanism responsible for these responses to celecoxib treatment in UC cells.

Autophagy is a cellular catabolic mechanism mediating the turnover of intracellular organelles and proteins through the lysosomal degradation. It's a principle mechanism to recycle the intracellular materials for energy production under stress [36], [46]. Autophagy has been reported to have paradoxical role in the control of cell death and survival [36]. While maintained at a basal level in resting cells, autophagy can be induced under conditions of stress and thus behaves as an adaptive survival mechanism [24]. SAPK/JNK and c-Jun, are MAPK-family signaling proteins that are activated by many types of cellular stress and functions to regulate a variety of cellular processes, including cell proliferation, differentiation, and apoptosis [47]. ER stress has been reported to induce autophagy [48], [49]. When misfolded proteins accumulate in the ER; the resulting stress activates the unfolded protein response to induce the expression of chaperones and proteins involved in the recovery process. The preautophagosomal structure is assembled, and subsequently transport of autophagosomes to the vacuole is stimulated in an Atg protein-dependent manner. As is shown in our results, induction of autophagy after celecoxib treatment may be associated with these stress responses.

In the process of autophagy, an autophagosome sequesters cytoplasmic constituents by forming a double-membrane vesicle. The outer membrane of autophagosome then fuses with lysosome to deliver the sequestered contents for degradation [46]. When autophagosome formation is activated, LC3B is increased; whereas lysosomal degradation of LC3B is simultaneously activated [40]. LC3B can be serves as a good indicator of autophagosome formation before they are destroyed through the fusion with lysosomes [40]. In this study, we found that celecoxib can trigger expression of LC3B, indicating the formation of autophagosome.

3-MA is a drug that selectively and potently inhibit autophagy-dependent protein degradation and suppress the formation of autophagosomes [43]. We found that 3-MA could effectively potentiate celecoxib-induced apoptosis in UC cells by suppressing the LC3B level and increasing caspases 3, 7 activations and PARP cleavage. Moreover, we found bafilomycin A1 also potentiated the celecoxib-induced apoptotic effect on UC cells. The increased expression of LC3B after clecoxib treatment could be due to either an increase in the flux of autophagosome formation or a reduction in the turnover of autophagosomes [36], [38]. Consistent with an effect of celecoxib in promoting the flux of autophagosomes, the presence of bafilomycin A1 led to a further increase in the levels of LC3B compared to that of celecoxib alone (data not shown). Thus, we assumed that celecoxib induced an increase in the autophagic activity. We could develop novel strategy to enhance the therapeutic efficacy of celecoxib.

The up-regulation of autophagy by GFP-tagged LC3B transfection can alleviate the celecoxib-induced cytotoxicity in UC cells. In mammalian cells, mTOR kinase, the target of rapamycin, mediates a major inhibitory signal on autophagy [36], [46]. Rapamycin, an mTOR inhibitor, is the most commonly utilized agent to mimic starvation-induced autophagy. Similarly, we found that the co-treatment of rapamycin significantly increased the level of LC3B and decreased celecoxib-induced apoptosis in UC cells. These findings indicated that upregulation of autophagy could decreases UC cell apoptosis induced by celecoxib.

Celecoxib is commonly administered orally with dosage of 200 mg twice daily, resulting in mean peak serum concentration of 1–2 µM [50]. Reported side effects of celecoxib in therapeutic dosage include cardiovascular thrombosis, congestive heart failure, gastrointestinal ulceration, renal or hepatic injury, and platelet aggregation [51]. Some reports on side effects of celecoxib in supra-therapeutic dosage in clinical trial showed that there were no significant side effects in supra-therapeutic dosage [52]. In the present study, we chose 80 µM as the working concentration of celecoxib, a concentration much higher than the concentration corresponding to the FDA recommended maximal dose. This is in line with a variety of studies on the anti-tumor effect of celecoxib *in vitro* showing that the concentration of celecoxib needed to inhibit growth of cancer cells *in vitro* is much higher than that needed *in vivo* for bladder cancer and other cancers [8]. This discrepancy indicates that tumor growth *in vivo* is determined by interactions between factors intrinsic to tumor cells and extrinsic factors such as the extracellular matrix, stromal cells, and other host factors. These extrinsic factors are generally absent under *in vitro* conditions. Cell culture models are often used to evaluate the therapeutic potential of COX-2 inhibitors against cancer, but it must be noted that *in vitro* results, particularly as relates to relative dose of agent used, cannot be directly extrapolated to the whole organism (*in vivo*) [53].

In conclusion, the present study showed that celecoxib could suppress the cell viability and induce autophagy in human UC cells. Autophagy inhibitor enhanced, but m-TOR inhibitor suppressed the cytotoxic efficacy of celecoxib in human UC cells. The regulation of autophagy suggests a novel therapeutic strategy against UC and warrants further study in the future.

## Supporting Information

Figure S1 **Celecoxib induces expression of stress-related molecules in human UC cells.** **(A)** NTUB1 and **(B)** T24 cells were treated with various concentrations of celecoxib for 24 h. Cell lysates were harvested at three time points (8, 16 and 24 h). We analyzed the effects of celecoxib on stress-related molecules of UC cells by Western blotting with specific antibodies to detect ER stress-related molecules (phospho-eIF2α and ATF-4) and other stress-related molecules (phospho-SAPK/JNK and phospho-c-Jun). Results shown are representative of at least three independent experiments.
(TIF)

Figure S2 **Celecoxib induces autophagy and apoptosis in a dose-dependent manner.** NTUB1 and T24 cells were treated with celecoxib in different concentrations (0, 20, 40, 60 and 80 µM) for 24 h. The cell lysates were analyzed by immunoblotting with antibodies against LC3B and cleaved caspase-7.
(TIF)

Figure S3 **Inhibition of autophagy by bafilomycin A1 and ATG7 knockdown potentiates celecoxib-induced cytotoxicity in human UC cells.** **(A)** NTUB1 and T24 cells were co-treated with celecoxib (80 µM) and bafilomycin A1 (5 nM) for 24 h. Apoptotic cells were analyzed by FACS flow cytometry with propidium iodide (PI) and annexin V-FITC staining. Data are presented as means ± SD. *: p<0.05 as statistically significant compared with celecoxib alone. **(B)** Cells were transfected with ATG7 siRNA (10 nM) or scramble siRNA (10 nM) (as a control); then treated with 80 µM celecoxib. The combinative effect of celecoxib and ATG7 knockdown on cell viability was determined by MTT assay and quantitative analysis was presented. Data are presented as means ± SD of three independents experiments. * p<0.05 as compared with scramble siRNA + celecoxib.
(TIF)

Figure S4 **The effects of LM-1685 on expressions of stress-related molecules, and LC3B activation in UC cells.** **(A)** NTUB1 **(B)** T24 cells were treated with LM-1685 (80 and 160 µM) or celecoxib (80 µM) for 24 h. The cell lysates were harvested and analyzed by Western blotting with specific antibodies to ATF-4, phospho-eIF2α and LC3B.
(TIF)

## References

[1] Jemal A, Siegel R, Xu J, Ward E (2010) Cancer statistics, 2010. CA Cancer J Clin 60: 277–300. 20610543 10.3322/caac.20073

[2] Latini DM, Lerner SP, Wade SW, Lee DW, Quale DZ (2010) Bladder cancer detection, treatment and outcomes: opportunities and challenges. Urology 75: 334–339. 19963252 10.1016/j.urology.2009.09.051

[3] Harker WG, Meyers FJ, Freiha FS, Palmer JM, Shortliffe LD, et al (1985) Cisplatin, methotrexate, and vinblastine (CMV): an effective chemotherapy regimen for metastatic transitional cell carcinoma of the urinary tract. A Northern California Oncology Group study. J Clin Oncol 3: 1463–1470. 4056840 10.1200/JCO.1985.3.11.1463

[4] Sternberg CN, Yagoda A, Scher HI, Watson RC, Herr HW, et al (1988) M-VAC (methotrexate, vinblastine, doxorubicin and cisplatin) for advanced transitional cell carcinoma of the urothelium. J Urol 139: 461–469. 3343727 10.1016/s0022-5347(17)42494-3

[5] Cohen MH, Rothmann M (2001) Gemcitabine and cisplatin for advanced, metastatic bladder cancer. J Clin Oncol 19: 1229–1231. 11181690 10.1200/JCO.2001.19.4.1229

[6] von der Maase H, Hansen SW, Roberts JT, Dogliotti L, Oliver T, et al (2000) Gemcitabine and cisplatin versus methotrexate, vinblastine, doxorubicin, and cisplatin in advanced or metastatic bladder cancer: results of a large, randomized, multinational, multicenter, phase III study. J Clin Oncol 18: 3068–3077. 11001674 10.1200/JCO.2000.18.17.3068

[7] Dannenberg AJ, Subbaramaiah K (2003) Targeting cyclooxygenase-2 in human neoplasia: rationale and promise. Cancer Cell 4: 431–436. 14706335 10.1016/s1535-6108(03)00310-6

[8] Dhawan D, Jeffreys AB, Zheng R, Stewart JC, Knapp DW (2008) Cyclooxygenase-2 dependent and independent antitumor effects induced by celecoxib in urinary bladder cancer cells. Mol Cancer Ther 7: 897–904. 18413803 10.1158/1535-7163.MCT-07-0313

[9] Dovedi SJ, Kirby JA, Atkins H, Davies BR, Kelly JD (2005) Cyclooxygenase-2 inhibition: a potential mechanism for increasing the efficacy of bacillus calmette-guerin immunotherapy for bladder cancer. J Urol 174: : 332–337; discussion 337. 10.1097/01.ju.0000161589.85869.ae 15947685

[10] Han C, Leng J, Demetris AJ, Wu T (2004) Cyclooxygenase-2 promotes human cholangiocarcinoma growth: evidence for cyclooxygenase-2-independent mechanism in celecoxib-mediated induction of p21waf1/cip1 and p27kip1 and cell cycle arrest. Cancer Res 64: 1369–1376. 14973068 10.1158/0008-5472.can-03-1086

[11] Maier TJ, Schilling K, Schmidt R, Geisslinger G, Grosch S (2004) Cyclooxygenase-2 (COX-2)-dependent and -independent anticarcinogenic effects of celecoxib in human colon carcinoma cells. Biochem Pharmacol 67: 1469–1478. 15041464 10.1016/j.bcp.2003.12.014

[12] Gee J, Lee IL, Jendiroba D, Fischer SM, Grossman HB, et al (2006) Selective cyclooxygenase-2 inhibitors inhibit growth and induce apoptosis of bladder cancer. Oncol Rep 15: 471–477. 16391871

[13] Grubbs CJ, Lubet RA, Koki AT, Leahy KM, Masferrer JL, et al (2000) Celecoxib inhibits N-butyl-N-(4-hydroxybutyl)-nitrosamine-induced urinary bladder cancers in male B6D2F1 mice and female Fischer-344 rats. Cancer Res 60: 5599–5602. 11059745

[14] Qin J, Yuan J, Li L, Liu H, Qin R, et al (2009) In vitro and in vivo inhibitory effect evaluation of cyclooxygenase-2 inhibitors, antisense cyclooxygenase-2 cDNA, and their combination on the growth of human bladder cancer cells. Biomed Pharmacother 63: 241–248. 18617357 10.1016/j.biopha.2008.04.007

[15] Gao M, Yeh PY, Lu YS, Hsu CH, Chen KF, et al (2008) OSU-03012, a novel celecoxib derivative, induces reactive oxygen species-related autophagy in hepatocellular carcinoma. Cancer Res 68: 9348–9357. 19010909 10.1158/0008-5472.CAN-08-1642

[16] Huang KH, Kuo KL, Chen SC, Weng TI, Chuang YT, et al (2012) Down-regulation of glucose-regulated protein (GRP) 78 potentiates cytotoxic effect of celecoxib in human urothelial carcinoma cells. PLoS One 7: e33615. 22438966 10.1371/journal.pone.0033615 PMC3306428

[17] Huang S, Sinicrope FA (2010) Celecoxib-induced apoptosis is enhanced by ABT-737 and by inhibition of autophagy in human colorectal cancer cells. Autophagy 6: 256–269. 20104024 10.4161/auto.6.2.11124 PMC2948490

[18] Jendrossek V, Handrick R, Belka C (2003) Celecoxib activates a novel mitochondrial apoptosis signaling pathway. FASEB J 17: 1547–1549. 12824303 10.1096/fj.02-0947fje

[19] Liu X, Yue P, Zhou Z, Khuri FR, Sun SY (2004) Death receptor regulation and celecoxib-induced apoptosis in human lung cancer cells. J Natl Cancer Inst 96: 1769–1780. 15572759 10.1093/jnci/djh322

[20] Pyrko P, Kardosh A, Liu YT, Soriano N, Xiong W, et al (2007) Calcium-activated endoplasmic reticulum stress as a major component of tumor cell death induced by 2,5-dimethyl-celecoxib, a non-coxib analogue of celecoxib. Mol Cancer Ther 6: 1262–1275. 17431104 10.1158/1535-7163.MCT-06-0629

[21] Kroemer G, Marino G, Levine B (2010) Autophagy and the integrated stress response. Mol Cell 40: 280–293. 20965422 10.1016/j.molcel.2010.09.023 PMC3127250

[22] Sato K, Tsuchihara K, Fujii S, Sugiyama M, Goya T, et al (2007) Autophagy is activated in colorectal cancer cells and contributes to the tolerance to nutrient deprivation. Cancer Res 67: 9677–9684. 17942897 10.1158/0008-5472.CAN-07-1462

[23] Jin S, White E (2007) Role of autophagy in cancer: management of metabolic stress. Autophagy 3: 28–31. 16969128 10.4161/auto.3269 PMC2770734

[24] Amaravadi RK, Thompson CB (2007) The roles of therapy-induced autophagy and necrosis in cancer treatment. Clinical cancer research : an official journal of the American Association for Cancer Research 13: 7271–7279. 18094407 10.1158/1078-0432.CCR-07-1595

[25] Hour TC, Chen J, Huang CY, Guan JY, Lu SH, et al (2000) Characterization of chemoresistance mechanisms in a series of cisplatin-resistant transitional carcinoma cell lines. Anticancer Res 20: 3221–3225. 11062746

[26] Yu HJ, Tsai TC, Hsieh TS, Chiu TY (1992) Characterization of a newly established human bladder carcinoma cell line, NTUB1. J Formos Med Assoc 91: 608–613. 1358347

[27] Huang AM, Kao YT, Toh S, Lin PY, Chou CH, et al (2011) UBE2M-mediated p27(Kip1) degradation in gemcitabine cytotoxicity. Biochem Pharmacol 82: 35–42. 21477582 10.1016/j.bcp.2011.03.026

[28] Hour TC, Lai YL, Kuan CI, Chou CK, Wang JM, et al (2010) Transcriptional up-regulation of SOD1 by CEBPD: a potential target for cisplatin resistant human urothelial carcinoma cells. Biochem Pharmacol 80: 325–334. 20385105 10.1016/j.bcp.2010.04.007 PMC3586239

[29] Cheng JH, Huang AM, Hour TC, Yang SC, Pu YS, et al (2011) Antioxidant xanthone derivatives induce cell cycle arrest and apoptosis and enhance cell death induced by cisplatin in NTUB1 cells associated with ROS. Eur J Med Chem 46: 1222–1231. 21345544 10.1016/j.ejmech.2011.01.043

[30] Lin KW, Huang AM, Yang SC, Weng JR, Hour TC, et al (2012) Cytotoxic and antioxidant constituents from Garcinia subelliptica. Food Chem 135: 851–859. 22868169 10.1016/j.foodchem.2012.04.133

[31] Li JR, Cheng CL, Yang CR, Ou YC, Wu MJ, et al (2013) Dual inhibitor of phosphoinositide 3-kinase/mammalian target of rapamycin NVP-BEZ235 effectively inhibits cisplatin-resistant urothelial cancer cell growth through autophagic flux. Toxicol Lett 220: 267–276. 23651616 10.1016/j.toxlet.2013.04.021

[32] Lee PC, Kakadiya R, Su TL, Lee TC (2010) Combination of bifunctional alkylating agent and arsenic trioxide synergistically suppresses the growth of drug-resistant tumor cells. Neoplasia 12: 376–387. 20454509 10.1593/neo.10110 PMC2864475

[33] Bubenik J, Baresova M, Viklicky V, Jakoubkova J, Sainerova H, et al (1973) Established cell line of urinary bladder carcinoma (T24) containing tumour-specific antigen. International journal of cancer Journal international du cancer 11: 765–773. 4133950 10.1002/ijc.2910110327

[34] Levine B, Kroemer G (2008) Autophagy in the pathogenesis of disease. Cell 132: 27–42. 18191218 10.1016/j.cell.2007.12.018 PMC2696814

[35] Suzuki SW, Onodera J, Ohsumi Y (2011) Starvation induced cell death in autophagy-defective yeast mutants is caused by mitochondria dysfunction. PLoS One 6: e17412. 21364763 10.1371/journal.pone.0017412 PMC3045454

[36] Xia HG, Zhang L, Chen G, Zhang T, Liu J, et al (2010) Control of basal autophagy by calpain1 mediated cleavage of ATG5. Autophagy 6: 61–66. 19901552 10.4161/auto.6.1.10326 PMC2883879

[37] Hanada T, Ohsumi Y (2005) Structure-function relationship of Atg12, a ubiquitin-like modifier essential for autophagy. Autophagy 1: 110–118. 16874032 10.4161/auto.1.2.1858

[38] Kabeya Y, Mizushima N, Ueno T, Yamamoto A, Kirisako T, et al (2000) LC3, a mammalian homologue of yeast Apg8p, is localized in autophagosome membranes after processing. The EMBO journal 19: 5720–5728. 11060023 10.1093/emboj/19.21.5720 PMC305793

[39] Degenhardt K, Mathew R, Beaudoin B, Bray K, Anderson D, et al (2006) Autophagy promotes tumor cell survival and restricts necrosis, inflammation, and tumorigenesis. Cancer Cell 10: 51–64. 16843265 10.1016/j.ccr.2006.06.001 PMC2857533

[40] Kabeya Y, Mizushima N, Ueno T, Yamamoto A, Kirisako T, et al (2000) LC3, a mammalian homologue of yeast Apg8p, is localized in autophagosome membranes after processing. EMBO J 19: 5720–5728. 11060023 10.1093/emboj/19.21.5720 PMC305793

[41] Kabeya Y, Mizushima N, Yamamoto A, Oshitani-Okamoto S, Ohsumi Y, et al (2004) LC3, GABARAP and GATE16 localize to autophagosomal membrane depending on form-II formation. J Cell Sci 117: 2805–2812. 15169837 10.1242/jcs.01131

[42] Schonthal AH (2009) Endoplasmic reticulum stress and autophagy as targets for cancer therapy. Cancer Lett 275: 163–169. 18692955 10.1016/j.canlet.2008.07.005

[43] Seglen PO, Gordon PB (1982) 3-Methyladenine: specific inhibitor of autophagic/lysosomal protein degradation in isolated rat hepatocytes. Proc Natl Acad Sci U S A 79: 1889–1892. 6952238 10.1073/pnas.79.6.1889 PMC346086

[44] Sakagami H, Kishino K, Amano O, Kanda Y, Kunii S, et al (2009) Cell death induced by nutritional starvation in mouse macrophage-like RAW264.7 cells. Anticancer research 29: 343–347. 19331171

[45] Ravikumar B, Vacher C, Berger Z, Davies JE, Luo S, et al (2004) Inhibition of mTOR induces autophagy and reduces toxicity of polyglutamine expansions in fly and mouse models of Huntington disease. Nat Genet 36: 585–595. 15146184 10.1038/ng1362

[46] Yorimitsu T, Klionsky DJ (2005) Autophagy: molecular machinery for self-eating. Cell death and differentiation 12 Suppl 21542–1552. 16247502 10.1038/sj.cdd.4401765 PMC1828868

[47] Kyriakis JM, Avruch J (2001) Mammalian mitogen-activated protein kinase signal transduction pathways activated by stress and inflammation. Physiol Rev 81: 807–869. 11274345 10.1152/physrev.2001.81.2.807

[48] Yorimitsu T, Klionsky DJ (2007) Endoplasmic reticulum stress: a new pathway to induce autophagy. Autophagy 3: 160–162. 17204854 10.4161/auto.3653

[49] Yorimitsu T, Nair U, Yang Z, Klionsky DJ (2006) Endoplasmic reticulum stress triggers autophagy. The Journal of biological chemistry 281: 30299–30304. 16901900 10.1074/jbc.M607007200 PMC1828866

[50] Davies NM, McLachlan AJ, Day RO, Williams KM (2000) Clinical pharmacokinetics and pharmacodynamics of celecoxib: a selective cyclo-oxygenase-2 inhibitor. Clin Pharmacokinet 38: 225–242. 10749518 10.2165/00003088-200038030-00003

[51] Menter DG, Schilsky RL, DuBois RN (2010) Cyclooxygenase-2 and cancer treatment: understanding the risk should be worth the reward. ClinCancer Res 16: 1384–1390. 10.1158/1078-0432.CCR-09-0788 PMC4307592 20179228

[52] Leese PT, Hubbard RC, Karim A, Isakson PC, Yu SS, et al (2000) Effects of celecoxib, a novel cyclooxygenase-2 inhibitor, on platelet function in healthy adults: a randomized, controlled trial. J Clin Pharmacol 40: 124–132. 10664917 10.1177/00912700022008766

[53] Williams CS, Watson AJ, Sheng H, Helou R, Shao J, et al (2000) Celecoxib prevents tumor growth in vivo without toxicity to normal gut: lack of correlation between in vitro and in vivo models. Cancer Res 60: 6045–6051. 11085526

