# Supplementary figures and images for: Celecoxib-Induced Cytotoxic Effect Is Potentiated by Inhibition of Autophagy in Human Urothelial Carcinoma Cells
Source: PLoS One. 2013 Dec 9;8(12):e82034. doi: 10.1371/journal.pone.0082034 (PMC3857231; doi:10.1371/journal.pone.0082034)

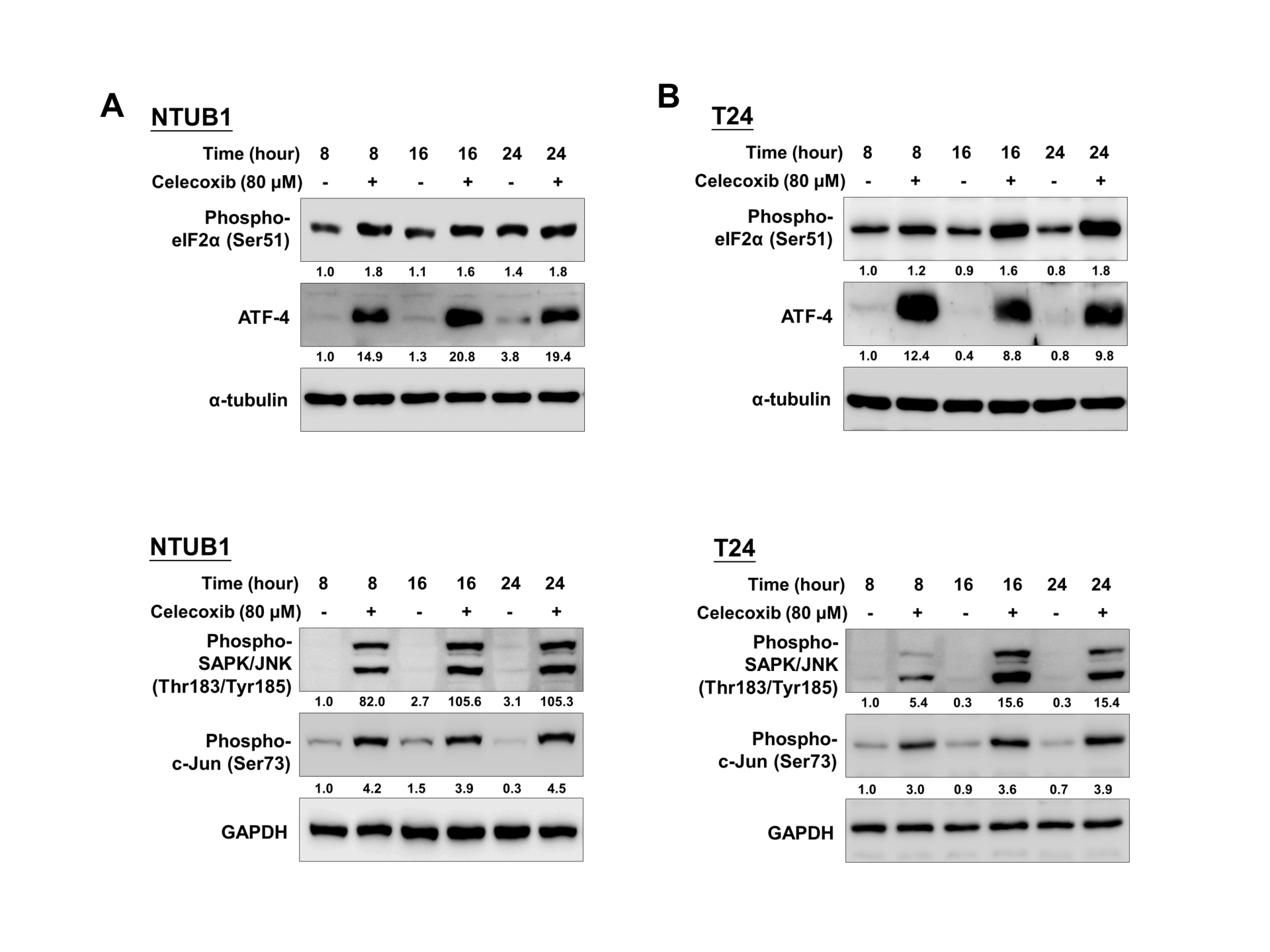

Supplement: Figure S1 — Celecoxib induces expression of stress-related molecules in human UC cells. (A) NTUB1 and (B) T24 cells were treated with various concentrations of celecoxib for 24 h. Cell lysates were harvested at three time points (8, 16 and 24 h). We analyzed the effects of celecoxib on stress-related molecules of UC cells by Western blotting with specific antibodies to detect ER stress-related molecules (phospho-eIF2α and ATF-4) and other stress-related molecules (phospho-SAPK/JNK and phospho-c-Jun). Results shown are representative of at least three independent experiments. (TIF) [file pone.0082034.s001.tif]

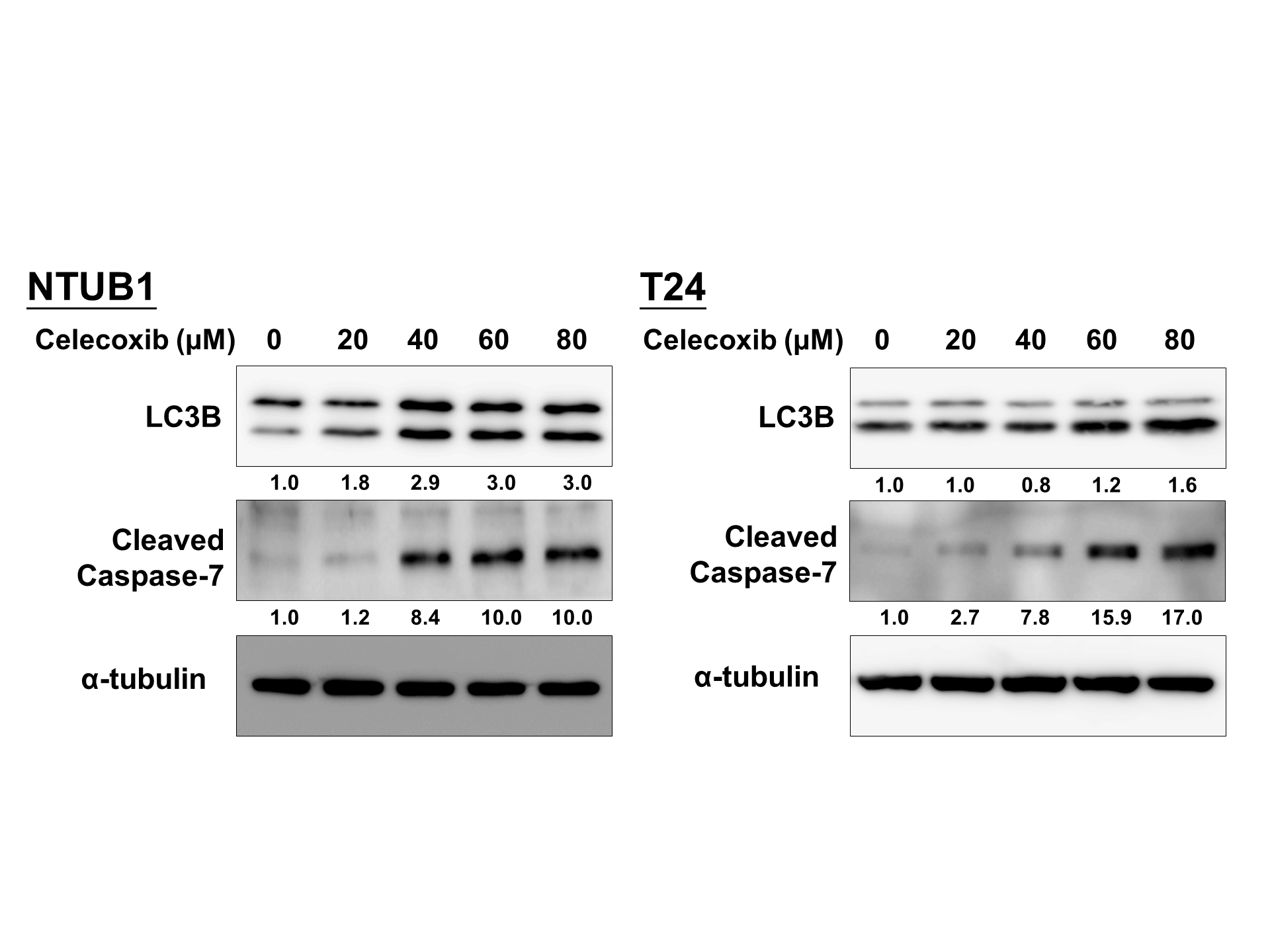

Supplement: Figure S2 — Celecoxib induces autophagy and apoptosis in a dose-dependent manner. NTUB1 and T24 cells were treated with celecoxib in different concentrations (0, 20, 40, 60 and 80 µM) for 24 h. The cell lysates were analyzed by immunoblotting with antibodies against LC3B and cleaved caspase-7. (TIF) [file pone.0082034.s002.tif]

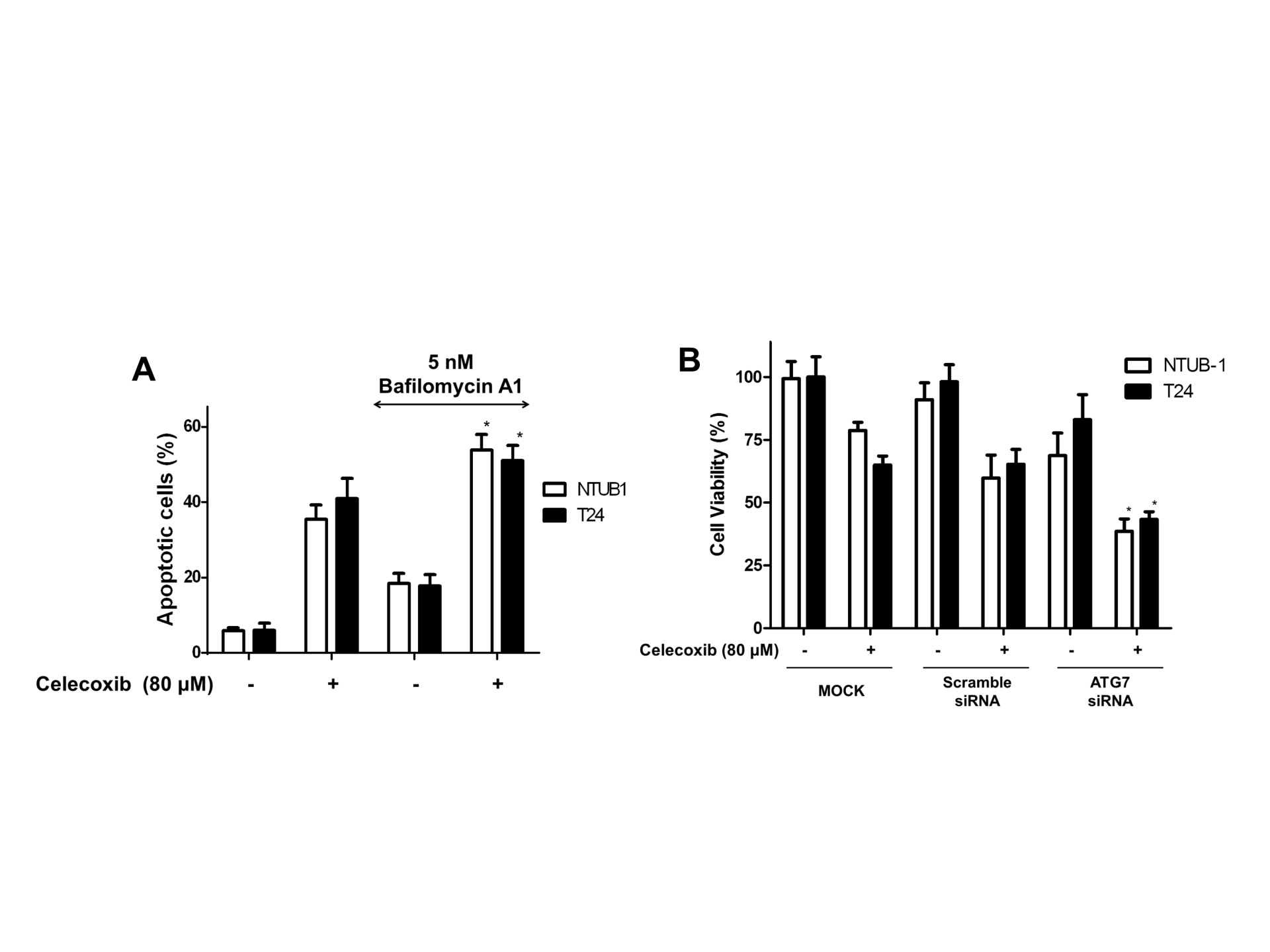

Supplement: Figure S3 — Inhibition of autophagy by bafilomycin A1 and ATG7 knockdown potentiates celecoxib-induced cytotoxicity in human UC cells. (A) NTUB1 and T24 cells were co-treated with celecoxib (80 µM) and bafilomycin A1 (5 nM) for 24 h. Apoptotic cells were analyzed by FACS flow cytometry with propidium iodide (PI) and annexin V-FITC staining. Data are presented as means ± SD. *: p<0.05 as statistically significant compared with celecoxib alone. (B) Cells were transfected with ATG7 siRNA (10 nM) or scramble siRNA (10 nM) (as a control); then treated with 80 µM celecoxib. The combinative effect of celecoxib and ATG7 knockdown on cell viability was determined by MTT assay and quantitative analysis was presented. Data are presented as means ± SD of three independents experiments. * p<0.05 as compared with scramble siRNA + celecoxib. (TIF) [file pone.0082034.s003.tif]

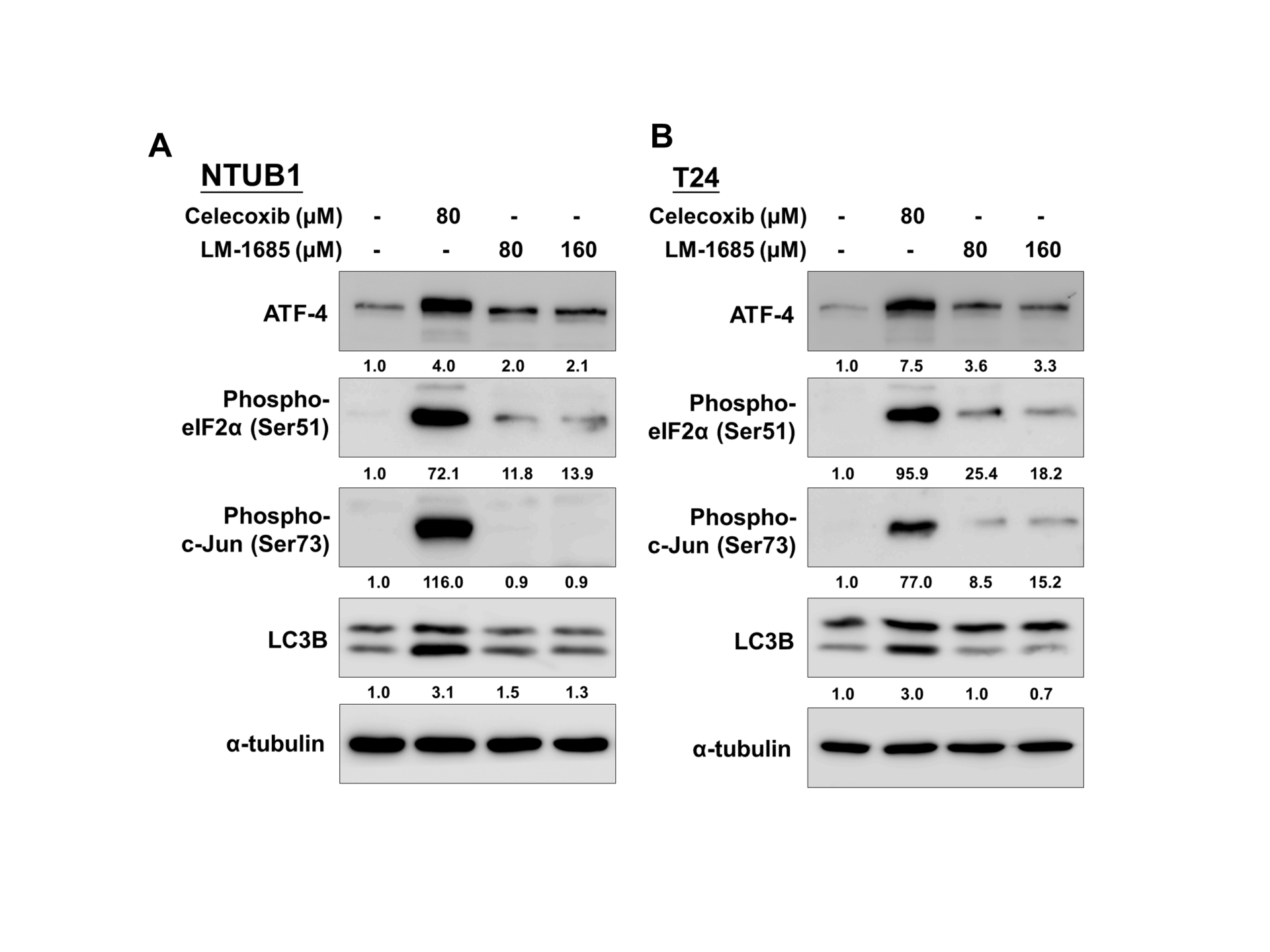

Supplement: Figure S4 — The effects of LM-1685 on expressions of stress-related molecules, and LC3B activation in UC cells. (A) NTUB1 (B) T24 cells were treated with LM-1685 (80 and 160 µM) or celecoxib (80 µM) for 24 h. The cell lysates were harvested and analyzed by Western blotting with specific antibodies to ATF-4, phospho-eIF2α and LC3B. (TIF) [file pone.0082034.s004.tif]
